# Acute kidney injury after cytoreductive surgery and hyperthermic intraperitoneal chemotherapy in patients with advanced ovarian cancer

**DOI:** 10.3389/fonc.2023.1094410

**Published:** 2023-01-25

**Authors:** Yun Bai, Ye Du, Pengpeng Ye, Yang Luo

**Affiliations:** ^1^ Department of Obstetrics and Gynecology, Beijing Jishuitan Hospital, Beijing, China; ^2^ Department of Medical Oncology, Beijing Chest Hospital, Capital Medical University & Beijing Tuberculosis and Tumor Research Institute, Beijing, China; ^3^ Division of Injury Prevention and Mental Health, National Center for Chronic and Non-communicable Disease Control and Prevention, Chinese Center for Disease Control and Prevention, Beijing, China; ^4^ Division of Nephrology, Beijing Shijitan Hospital, Capital Medical University, Bejing, China

**Keywords:** ovarian cancer, acute kidney injury, cytoreductive surgery, hyperthermic intraperitoneal chemotherapy, risk factor

## Abstract

**Background:**

Ovarian cancer is one of the most common gynecologic cancers with the highest mortality rate in China. Acute kidney injury (AKI) is a postoperative complication associated with all-cause mortality. The incidence and risk factors for AKI after cytoreductive surgery with hyperthermic intraperitoneal chemotherapy (CRS-HIPEC) have not been fully elucidated. The purpose of this study was to determine the incidence and associate ed risk factors of AKI among those patients undergoing CRS-HIPEC.

**Methods:**

This retrospective study collected demographic, tumor-related, preoperative, intraoperative, and postoperative data from 282 advanced ovarian cancer patients who underwent CRS-HIPECs. AKI was defined and staged according to the clinical practice guideline of Kidney Disease Improving Global Outcomes (KDIGO) in 2012. The prognosis of AKI was determined according to the change in serum creatinine 90 days after the operation. We conducted univariate and multivariate logistic regression analyses to assess the association between variables of interest and the occurrence of AKI.

**Results:**

Of 282 advanced ovarian cancer patients, 11.7% of them developed AKI. The Multivariate logistic regression analysis showed that the risk factors independently associated with AKI included cisplatin dose≥70mg/m^2^ (OR=3.668, 95%CI 1.336-10.070, P=0.012); Baseline eGFR<60 mL/min/1.73 m^2^ (OR=2.704, 95%CI 1.373-5.322, P=0.004); and concomitant medications of angiotensin convert enzyme inhibitor or angiotensin receptor blocker (ACEI or ARB) (OR=3.122, 95%CI 1.545-14.892, P=0.039).

**Conclusion:**

Our study demonstrates that the incidence of AKI after CRS plus cisplatin-based HIPEC is not uncommon among advanced ovarian cancer patients. Cisplatin overdose, baseline kidney dysfunction, and use of ACEI or ARB are independent risk factors for the occurrence of AKI among those patients.

## Introduction

Ovarian cancer has become one of the most common gynecologic cancers with the highest mortality rate in China (1, 2). As it always grows secretly, the onset symptoms of ovarian cancer are often non-specific and easy to be overlooked at its early stage, about 70% of patients with ovarian cancer are diagnosed at advanced stages (3). Currently, cytoreductive surgery (CRS) plus hyperthermic intraperitoneal chemotherapy (HIPEC) has become a promising strategy for advanced ovarian cancer (4, 5). A recent randomized control study showed that the addition of HIPEC to interval CRS resulted in longer recurrence-free survival and overall survival than surgery alone among patients with stage III epithelial ovarian cancer (6).

However, there are also data indicating that the application of CRS plus HIPEC has some non-eligible complications, and most causes of systemic toxicity of it are related to renal toxicity and bone marrow failure (7, 8). The occurrence of acute kidney injury (AKI) in patients undergoing CRS plus HIPEC was also reported, and the incidence of it varied widely between 1% and 48%, the reason for the great variability in the prevalence of AKI might be related to different study populations, different regimens and doses for chemotherapy, besides these, the application of different definitions of AKI was also an important reason for that wide range of the AKI occurrence (9). Meanwhile, some associated risk factors for the occurrence of AKI like increased age, obesity, intraoperative blood loss, baseline renal dysfunction, preoperative hypoalbuminemia, and use of angiotensin-II receptor antagonists were identified among those patients (10–12). As the application of CRS plus HIPEC among advanced ovarian cancer patients developed so quickly in the last decades, it seems to be necessary to make a further evaluation of the incidence of AKI and associated risk factors in those patients undergoing CRS-HIPEC treatment. Although CRS plus HIPEC has been introduced to advanced ovarian cancer patients for more than 10 years in China, there is still no systemic assessment of AKI-related complications among Chinese ovarian cancer patients (6).

Our study aimed to assess the incidence and identify the associated risk factors of AKI in a group of Chinese advanced ovarian cancer patients undergoing the CRS plus cisplatin-based HIPEC.

## Methods

### Study design and participants

This is a retrospective cohort study. Patients who were consecutively diagnosed with International Federation of Gynecology and Obstetrics (FIGO) stage IIIC and IV epithelial ovarian cancer (including peritoneal, ovarian, and fallopian tube cancers) and received the CRS plus cisplatin-based HIPEC were included in the database of Beijing Shijitan Hospital, Capital Medical University between January 2018 and December 2021. The patients who had confirmed end-stage renal disease before the CRS plus cisplatin-based HIPEC or who did not finish the whole process of the therapy were excluded. The ethical approval for this study was granted by the Institutional Ethical Review Board of Beijing Shijitan Hospital, Capital Medical University [sjtkyll-lx-2020-35]). The informed consent for inclusion in the present study was waived because it was a retrospective study and only de-identified information was used in this study.

### Cytoreductive surgery and HIPEC

The cytoreductive surgery was performed following Sugarbaker principles of the peritonectomy (13). The main goal of the CRS was to obtain complete cytoreduction, which was evaluated by applying the Completeness Cytoreduction Score, it was classified into three levels depending on the number of visceral resections and peritonectomy procedures (level I, one or two procedures; level II, three or four procedures; level III, five or more procedures) (14, 15). The CRS was followed by cisplatin-based HIPEC, which was performed according to the Chinese expert consensus on CRS plus HIPEC for peritoneal malignancies, cisplatin was given at a dose from 50 to 80 mg/m^2^, heated at between 41°C and 43°C for 60 min, and using an open coliseum technique, the regimens of HIPEC therapy for individual patients were mainly determined by consultant surgeons, and cisplatin dose over 70mg/m^2^ was regarded as a high dose of cisplatin therapy in the HIPEC treatment (16). All patients had previously received platinum-based chemotherapy. Patients had received a maximum of six cycles in frontline treatment while those with recurrent ovarian cancer received a maximum of 12 cycles.

### Data collection

The data were retrieved from the patient’s electronic medical charts in the database of our hospital. The medical information of each patient was reviewed and recorded, including demographic information, comorbidities (hypertension, diabetes mellitus, dyslipidemia, and coronary heart disease [CHD]), concomitant medications 7 days before operation (angiotensin-converting enzyme inhibitors [ACEI] or angiotensin receptor blockers [ARB], diuretics, non-steroidal anti-inflammatory drugs [NSAIDs], proton pump inhibitor [PPI]), body mass index (BMI), systolic blood pressure (SBP), diastolic blood pressure (DBP), previous oncological related treatments (chemotherapy or chemotherapy plus surgery), American Society of Anesthesiologists (ASA) stages, operative details (Peritoneal Carcinomatosis Index [PCI] score, the number of visceral resections, level of peritonectomy and drug dose of HIPEC perfusion, ascitic volumes, operative time, estimated blood loss volumes and hypotension). We also collected data on preoperative serum concentrations of hemoglobin and albumin. The baseline eGFR was calculated by using the Chronic Kidney Disease Epidemiology Collaboration (CKD-EPI) creatinine equation with an adjusted coefficient of 1.1 for the Asian population and categorized as ≥60 and <60 ml/min/1.73 m^2^ (17). Serum creatinine (Scr) before, 1,2, 7 days, and 90 days after CRS plus cisplatin-based HIPEC were also retrieved from the medical records.

### Diagnosis of AKI

AKI was defined and staged according to the clinical practice guideline of Kidney Disease Improving Global Outcomes (KDIGO) in 2012 (AKI stage I: increase in Scr>0.3 mg/dl within 48 h, or increase to at least 1.5 times baseline within the prior 7 days; stage II: increase in Scr 2-2.9 times baseline; stage III: increase in Scr >4 mg/dl or greater than 3 times of the baseline or receiving renal replacement therapy) (18). Depending on the abovementioned definition of AKI, we analyzed Scr on postoperative days 0,1, 2, and 7 after the treatment. Indications for renal replacement therapy were set by an interdisciplinary consultant.

We defined renal recovery as full recovery with Scr decreased to below threshold or to the baseline, partial recovery as serum creatinine decreased by 25% or more from peak concentration but remained higher than the threshold or baseline, and non-recovery as the patient still dependent on dialysis or serum creatinine decreased by less than 25% from peak concentration within 90 days following AKI (19).

### Statistical analysis

Continuous variables were expressed as mean with standard deviation (SD) or median with interquartile range (IQR); categorical variables were expressed as a number with a percentage. Differences between the AKI group and non-AKI group were compared using Chi-square tests for categorical variables and student’s t-test or Mann-Whitney U test, as appropriate, for continuous variables. We used univariate and multivariate logistic regression analysis to evaluate factors related to AKI, with AKI as the dependent variable, and all baseline variables as covariates, including age, comorbidities (hypertension, diabetes mellitus, dyslipidemia, and CHD), concomitant medications (ACEI or ARB, diuretics, NSAIDs, PPI), previous oncological treatments (chemotherapy or chemotherapy plus surgery, BMI, SBP, DBP, serum concentration of hemoglobin, albumin, and baseline GFR<60 ml/min/1.73m^2^, as well as ASA stages, some operative parameters, such as PCI score, number of visceral resections, level of peritonectomy, ascitic volumes, operative time, estimated blood loss volumes and hypotension, and chemotherapy regimen of HIPEC (we stratified our patients into two groups by the dose of cisplatin at 70mg/m^2^). All covariates with a P value of less than 0.10 on univariable analysis were entered into the multivariable model. We reported an odds ratio (OR) with a 95% confidence interval (95% CI) for each covariate of interest. Statistical significance was set at a value of p<0.05. Analyses were performed with SPSS version 21.0 statistical software (SPSS Inc, Chicago, IL, USA).

## Results

### Sociodemographic and clinical characteristics

We first enrolled 296 patients from the database of our hospital, 14 patients were excluded according to the exclusion criteria, and we finally included 282 patients in our study. AKI occurred in 11.7% of patients (n = 33), while 88.3% (n = 249) were non-AKI patients. Patients in the AKI group were more likely to have comorbid hypertension and diabetes mellitus, concomitant medications of ACEI or ARB (%), previous treatment of chemotherapy plus surgery, and a higher proportion of baseline eGFR <60 ml/min/1.73m^2^ (P<0.05 or P<0.001). Regarding intraoperative parameters, patients in the AKI group were more likely to have more visceral resections and estimated blood loss, and a higher proportion of cisplatin dose ≥70 mg/m^2^ compared with the non-AKI group (P<0.05). There were no significant differences in age, BMI, blood pressure, ASA stages, baseline serum level of hemoglobin and albumin, and some intraoperative parameters (including PCI scores, peritonectomy procedures, operation time, proportions of ascites >500ml and hypotension) between the two groups (P>0.05) (**Table 1**).

**Table 1:** Demographics and operative characteristics of patients.

Characteristics	All (n = 282)	Non- AKI (n = 249)	AKI (n = 33)	*P-*value
Preoperative parameters				
Age (years)	64.83 ± 7.83	64.80 ± 7.99	65.03 ± 6.63	0.876
Comorbidities, n (%)				
Hypertension	105 (37.2)	87 (24.9)	18 (54.6)	**0.029**
Diabetes mellitus	44 (15.6)	34 (13.7)	10 (30.3)	**0.013**
Dyslipidemia	31 (11.0)	27 (10.8)	4 (12.1)	0.825
CHD	28 (9.9)	25 (10.0)	3 (9.1)	0.864
Concomitant medications, n (%)				
ACEI or ARB	34 (12.1)	25 (10.0)	9 (27.3)	**0.004**
Diuretics	32 (11.3)	26 (10.4)	6 (18.2)	0.188
NSAIDs	229 (81.2)	201 (80.7)	28 (84.8)	0.569
PPI	54 (19.1)	48 (19.3)	6 (18.2)	0.909
ASA stage, n (%)	0.138			
I–II	162 (57.4)	147 (59.0)	15 (44.5)	
III–IV	120 (42.6)	102 (41.0)	18 (54.5)	
Type of treatment, n (%)	0.019			
Frontline	181 (64.2)	173 (69.5)	8 (24.2)	
Recurrence	101 (35.8)	76 (30.5)	25 (75.8)	
Previous treatment, n (%)	0.020			
Chemotherapy + surgery	118 (41.8)	98 (39.4)	20 (60.6)	
Chemotherapy	164 (58.2)	151 (60.6)	13 (39.4)	
BMI (kg/m^2^)	26.91 ± 3.27	26.84 ± 3.19	27.48 ± 3.77	0.288
SBP (mmHg)	127.39 ± 13.26	127.25 ± 12.88	128.45 ± 16.06	0.626
DBP (mmHg)	77.05 ± 9.35	76.69 ± 8.64	79.73 ± 13.42	0.080
Hb (g/L)	106.14 ± 18.21	106.80 ± 18.09	101.12 ± 18.62	0.092
ALB (g/L)	38.00 ± 3.83	38.05 ± 3.91	37.68 ± 3.20	0.601
eGFR (ml/min/1.73m^2^)	101.46 ± 26.56	102.95 ± 24.35	92.80 ± 36.02	0.089
eGFR <60 ml/min/1.73m^2^, n (%)	28 (9.9)	20 (8.0)	8 (24.2)	0.003
Intra-operative parameters				
PCI score, n (%)				0.521
<8	143 (50.7)	128 (51.4)	15 (45.5)	
≥8	139 (49.3)	121 (48.6)	18 (54.5)	
Visceral resections, n (%)	0.005			
0	116 (41.1)	106 (42.6)	10 (30.3)	
1-2	145 (51.4)	129 (51.8)	16 (48.5)	
≥3	21 (7.4)	14 (5.6)	7 (21.2)	
Peritonectomy procedures, n (%)	0.138			
0	47 (16.7)	45 (18.1)	2 (6.1)	
Level I	88 (31.2)	76 (30.5)	12 (36.4)	
Level II	76 (27.0)	57 (22.9)	5 (15.2)	
Level III	71 (25.2)	71 (28.5)	14 (42.4)	
Cisplatin dose, n (%)	0.001			
<70 mg/m^2^	229 (81.2)	209 (83.9)	20 (60.6)	
≥70 mg/m^2^	53 (18.8)	40 (16.1)	13 (39.4)	
Operation time (min)	178.86 ± 60.90	177.41 ± 58.35	189.79 ± 77.70	0.273
Estimated blood loss (ml)	240.0 (180.0, 490.0)	230.0 (170.0, 480.0)	300.0 (210.0, 680.0)	0.048
Ascites >500ml, n (%)	11 (3.9)	9 (3.6)	2 (6.1)	0.495
Hypotension (%)	21 (7.4)	16 (6.4)	5 (15.2)	0.073

Values are shown as mean ± standard deviation, median with interquartile range or n (%).

Bold values represent P < 0.05.

AKI, acute kidney disease; CHD, coronary heart disease; ACEI, angiotensin-converting enzyme inhibitors; ARB, angiotensin receptor blockers; NSAIDs, non-steroidal anti-inflammatory drugs; PPI, proton pump inhibitor; BMI, body mass index; SBP, systolic blood pressure; DBP, diastolic blood pressure; Hb, hemoglobin; ALB, albumin; eGFR, estimated glomerular filtration rate; ASA, American Society of Anesthesiologists; PCI, Peritoneal Carcinomatosis Index.

### AKI stages and renal recovery

Among the patients who developed AKI, the majority of them were in mild-to-moderate severity (KDIGO stage 1: 16 (48.5% of AKI episodes), stage 2: 11 (33.3%), and stage 3: 6 (18.2%)), and only 1 patient received acute renal replacement therapy, she was 58 years old woman whose baseline eGFR was 51mL/min/1.73 m^2^, she also had diabetes and hypertension, ARB was used to control the blood pressure, her HIPEC treatment dose of cisplatin was 60 mg/m^2^. The median time from surgery initiation to the occurrence of AKI was 3.5 (IQR 2.5 to 6.5) days. Of the 33 patients with AKI, full renal recovery was achieved in 10 (30.3%) patients, partial recovery in 14 (42.4%) patients, and failure to recover in 9 (27.3%) patients until 90 days after AKI.

### Associated risk factors for AKI

The results of univariate logistic regression were listed in **Table 2**, AKI after CRS-HIPEC was associated with comorbidities of hypertension and diabetes mellitus, concomitant medications of ACEI or ARB, previous chemotherapy + surgery, Baseline eGFR<60 mL/min/1.73 m^2^ visceral resections≥3, cisplatin dose≥70mg/m^2^, intraoperative blood loss and hypotension(p<0.10); Multivariate logistic regression analysis showed that the risk factors independently associated with AKI included concomitant medications of ACEI or ARB (OR=3.122, 95%CI 1.545-14.892, P=0.039), Baseline eGFR<60 mL/min/1.73 m^2^ (OR=2.704, 95%CI 1.373-5.322, P=0.004), and cisplatin dose≥70mg/m^2^ (OR=3.668, 95%CI 1.336-10.070, P=0.012) (**Table 3**).

**Table 2:** Univariate analysis of risk factors for AKI after CRS plus HIPEC.

Variables	Unadjusted OR	95% CI	*P*-value
Comorbidities			
Hypertension	2.665	1.207-12.134	**0.032**
Diabetes Mellitus	3.255	1.254-12.009	**0.015**
ACEI or ARB	3.589	1.640-14.840	**0.004**
Previous chemotherapy + surgery	2.967	1.018-10.764	0.028
Visceral resections			
0	1.000		
1-2	1.114	0.275-4.507	0.880
≥3	4.612	1.364-23.089	**0.017**
Cisplatin dose ≥70 mg/m^2^	4.424	1.559-10.522	**0.002**
Intraoperative blood loss (each 100 ml)	1.172	0.940-1.321	0.051
Intraoperative hypotension	4.756	0.985-17.950	0.075

Bold values represent P < 0.05.

AKI, acute kidney disease; CRS, cytoreductive surgery; HIPEC, hyperthermic intraperitoneal chemotherapy; ACEI, angiotensin-converting enzyme inhibitors; ARB, angiotensin receptor blockers; eGFR, estimated glomerular filtration rate.

**Table 3:** Multivariate analysis of risk factors for AKI after CRS plus HIPEC.

Variables	Adjusted OR	95% CI	*P*-value
Comorbidities			
Hypertension	2.614	0.654 – 10.447	0.274
Diabetes Mellitus	2.854	0.818 –10.764	0.198
ACEI or ARB	3.122	1.545 – 14.892	**0.039**
Previous chemotherapy + surgery	2.887	0.875 – 11.023	**0.164**
Baseline eGFR<60 mL/min/1.73 m^2^	2.704	1.373 – 5.322	**0.004**
Visceral resections			
0	1.000		
1-2	1.205	0.324 – 4.132	0.856
≥3	4.422	0.902 – 20.345	0.088
Cisplatin dose ≥70 mg/m^2^	3.668	1.336 – 10.070	**0.012**
Intraoperative blood loss (each 100 ml)	1.050	0.754 – 1.502	0.367
Intraoperative hypotension	3.567	0.906 –15.764	0.118

All covariates with a P value of less than 0.10 on univariable analysis were entered into the multivariable model, including comorbidities of diabetes mellitus and hypertension, combined ACEI or ARB, previous chemotherapy + surgery, baseline eGFR<60 ml/min/1.73m^2^, visceral resections, cisplatin dose ≥70 mg/m^2^, intraoperative blood loss, and intraoperative hypotension.

Bold values represent P < 0.05.

AKI, acute kidney disease; CRS, cytoreductive surgery; HIPEC, hyperthermic intraperitoneal chemotherapy; ACEI, angiotensin-converting enzyme inhibitors; ARB, angiotensin receptor blockers; eGFR, estimated glomerular filtration rate.

## Discussion

In this retrospective study, we found that the incidence of AKI was 11.7% in a group of Chinese advanced ovarian cancer patients undergoing the CRS plus cisplatin-based HIPEC. A high dose of cisplatin, baseline kidney dysfunction, and the use of ACEI or ARB were independent risk factors for the occurrence of AKI. By applying the KDIGO definition of AKI, our results determined some important risk factors in the early identification of AKI in advanced ovarian cancer patients undergoing CRS plus cisplatin-based HIPEC treatment. To the best of our knowledge, our study was also the first related report in the area of the association of CRS plus HIPEC and the occurrence of AKI among Chinese advanced ovarian cancer patients.

As one of the serious complications in cancer patients, AKI has been proven to be associated with increased all-cause mortality and received great attention in clinical settings in recent years (20, 21). Previous studies showed that the incidence of AKI after CRS plus HIPEC ranged from 1 to 48% (9). This vast variability might be related to the different ethnicities of the study population, and different chemotherapy protocols and doses applied in the treatment, besides, lacking consensus criteria of AKI might be another important reason for such a large variability of the incidence of AKI among those patients (22). Some previous analyses used a three-fold increase of Scr as the criteria for identifying AKI, the incidence of AKI in those studies was only 1.3 to 5.7%, under this condition, some existing AKI episodes could not meet that criteria, and the results of the AKI incidence in these studies might be therefore underestimated (23–25). Compared with the above-mentioned studies, Angeles et al. (26) identified AKI with Risk, Injury, Failure, Loss, and End-stage kidney function (RIFLE) criteria, which is the first international interdisciplinary consensus criteria for diagnosis of AKI, the incidence of AKI in a group of ovarian peritoneal carcinomatosis with RIFLE criteria was increased to 48%, this results might be explained by the strictness of AKI diagnosis criteria which included some small changes of kidney function during the treatment, at the same time, all patients in that study had previously received systemic platinum-based chemotherapy for at least 6 cycles, which might have caused basic kidney injury and became a high-risk group of patients of being developed to AKI. Currently, the KDIGO criteria of AKI was recommended by the International Kidney Foundation, compared with the RIFLE criteria, it could be more sensitive in identifying AKI by capturing smaller changes in Scr (>0.3 mg/dl within 48 h) among the patients (27, 28). In our study, we applied the KDIGO criteria to identify AKI, and the incidence of AKI was much lower than that in Angeles’ report, the reason might be related to the different selection criteria of the ovarian cancer patients, in our study, only cisplatin was applied in HIPEC, and the average operation time was shorter than that in Angeles’ study (60 mins vs. 90 mins). The discrepancy in the incidence of AKI reminded us that we should pay attention to the diagnostic criteria of AKI applied in the study and the patient’s clinical features (like cancer stage and systemic chemotherapy regimen) while analyzing those related data. At the same time, it would be more comparable on the same issue if future studies could use the KDIGO criteria of AKI.

Another concern in our study was to identify the potential risk factors of AKI in advanced ovarian cancer patients while receiving CRS plus HIPEC. It has been proposed that most episodes of AKI in cancer patients are closely related to kidney parenchymal ischemia and tubal epithelial intoxication (29). Previous studies showed that increased age, obesity, baseline serum creatinine, and use of ACEI or ARB were independent risk factors associated with the occurrence of AKI in those advanced ovarian cancer patients, however, the risk factors in different studies were still inconsistent (5). Although the systemic application of cisplatin has been identified as an independent risk factor for AKI in different cohorts of ovarian cancer patients, the role of cisplatin in the development of AKI in HIPEC therapy was still not determined in the previous study, some studies failed to identify cisplatin as an independent risk factor for HIPEC-induced AKI (30). To make it clear, our study selected ovarian cancer patients who received HIPEC with a single regimen of cisplatin, the doses of cisplatin in our study ranged from 50 to 80 mg/m^2^. In a multicenter phase I study, the nephrotoxicity would be increased significantly if the dose of cisplatin was greater than 70mg/m^2^, this dose of cisplatin was also regarded as a high dose for HIPEC treatment in the Chinese expert consensus on CRS plus HIPEC for peritoneal malignancies (16, 31). To validate the renal toxicity of this dose of cisplatin, we stratified our patients into two groups by the dose of cisplatin at 70mg/m^2^ in the treatment, our results showed that the risk of developing AKI was increased significantly when the dose of cisplatin was more than 70mg/m^2^, this data suggested that the nephrotoxicity of cisplatin appeared to be dose-dependent manner, and the dose of cisplatin should be adjusted according to the conditions of individual patients, at the same time, the kidney function during and after the HIPEC therapy process should also be monitored closely.

Additionally, our study also showed that baseline eGFR less than 60 mL/min/1.73 m^2^ was an independent risk factor for the occurrence of AKI, this was consistent with a study from Singapore, which included 47 advanced ovarian cancer patients undergoing CRS plus HIPEC treatment (32). In most situations, increased baseline Scr level or decreased eGFR often reflects the existence of chronic kidney disease that is related to renal parenchymal ischemia and injury in some systemic diseases like hypertension or diabetes, it is regarded that more than 60% of kidney function has been lost at the time when eGFR is less than 60 mL/min/1.73 m^2^, and we should pay more attention to those patients with increased baseline Scr or decreased eGFR before start CRS plus cisplatin-based HIPEC treatment (29, 33). Our result also indicated that the application of ACEI or ARB was independently associated with the occurrence of AKI, similar to our study, Hakeam et al. (34) reported an association of angiotensin II receptor blocker use and hypertension with postoperative AKI in patients undergoing CRS plus HIPEC therapy. In the animal model of AKI, both protective and aggravating effects of ACEI or ARB have been identified, but underlying mechanisms remained elusive, and the potential nephrotoxic effect of them in acute settings might be related to renal parenchymal ischemia which was aggravated by ACEI or ARB (33). These results remind us to pay more attention to those related risk factors and try to identify the occurrence of AKI.

The main strength of our study was the application of the KDIGO criteria in the diagnosis of AKI, which has been validated as one of the most sensitive criteria in identifying AKI, widely applying this definition of AKI could make future studies to be comparable in the future. Our study also has some limitations that need to be addressed. First, our study was a single-center observational study, we cannot adjust all of the residuals and unmeasured confounding. Those potential existing risk factors should be added in future analyses. However, a single-center study has some advantages of the similarity of the quality control in the whole process of the study. Secondly, as a retrospective observational study, our results should be carefully extrapolated to other ovarian cancer patients of different ethnicity, as some potential risk factors like pharmaco-ethnicity of different susceptibility to chemotherapeutic drugs may also be meaningful among different ethnic groups of patients, these issues also need to be identified in the future study.

## Conclusions

Our present study indicated that the incidence of acute kidney injury after CRS plus cisplatin-based HIPEC is not uncommon among advanced ovarian cancer patients. High doses of cisplatin, baseline kidney dysfunction, and the use of ACEI or ARB are independent risk factors for the occurrence of AKI among those patients. Future studies should focus on establishing a clear protocol for patients undergoing CRS plus HIPEC to reduce acute kidney injury.

## Data availability statement

The original contributions presented in the study are included in the article/supplementary material. Further inquiries can be directed to the corresponding author.

## Ethics statement

The studies involving human participants were reviewed and approved by The Institutional Ethical Review Board of Beijing Shijitan Hospital, Capital Medical University [sjtkyll-lx-2020-35]). Written informed consent for participation was not required for this study in accordance with the national legislation and the institutional requirements.

## Author contributions

YB and YL designed the study. YD and PY collected data and performed the statistical analysis, YB and YD and wrote the manuscript draft. All authors contributed to data interpretation and editing of the final manuscript and approved the final version of the manuscript.
